# Open-Label Placebo Administration Decreases Pain in Elderly Patients With Symptomatic Knee Osteoarthritis – A Randomized Controlled Trial

**DOI:** 10.3389/fpsyt.2022.853497

**Published:** 2022-05-06

**Authors:** Elisabeth Olliges, Sabine Stroppe, Anja Haile, Fabienne Reiß, Marwa Malhis, Susanne A. Funke, Karin Meissner

**Affiliations:** ^1^Medical Faculty, Institute of Medical Psychology, Ludwig Maximilian University of Munich, Munich, Germany; ^2^Division of Health Promotion, Coburg University of Applied Sciences and Arts, Coburg, Germany; ^3^Institute for Bioanalysis, Coburg University of Applied Sciences and Arts, Coburg, Germany

**Keywords:** chronic pain, knee arthrosis, open-label placebo, placebo effect, mood, verbal instruction

## Abstract

**Background:**

Recent studies indicate that the administration of open-label placebos (OLP) can improve symptoms in various medical conditions. The primary aim of this 3-week randomized controlled trial was to examine the effects of OLP treatments on pain, functional disability, and mobility in patients with arthritic knee pain.

**Methods:**

Sixty patients (55% females; mean age, 66.9 ± 9.7 SD years) were randomized to one of two OLP treatments (*n* = 41) or no treatment (NT; *n* = 19). OLP treatments were accompanied by the verbal suggestion “to decrease pain” (OLP-pain, *n* = 20) or “to improve mood” (OLP-mood, *n* = 21). Pain and mood levels were monitored on 11-point Numeric Rating Scales (NRSs) in a patient diary, and global clinical improvement (CGI-I) was assessed at the end of the study. At baseline and after 21 days, patients filled in validated questionnaires to assess symptoms and functional disability of the knee (WOMAC), mental and physical quality of life (SF-36), state anxiety (STAI-state), perceived stress (PSQ-20), and self-efficacy (GSE). In addition, knee mobility (neutral zero-method), heart rate variability (HRV), and diurnal cortisol levels were evaluated before and after treatment.

**Results:**

Evaluation of daily pain ratings indicated significant pain decrease in the OLP groups compared to NT (*p* = 0.013, *d* = 0.64), with no difference between the OLP-pain and the OLP-mood groups (*p* = 0.856, *d* = 0.05). OLP treatment also improved WOMAC pain (*p* = 0.036, *d* = 0.55), again with no difference between the two OLP groups (*p* = 0.65, *d* = 0.17). WOMAC function and stiffness, knee mobility, stress, state anxiety, quality of life, and self-efficacy did not change differently between groups.

**Conclusion:**

OLP treatment improved knee pain in elderly patients with symptomatic knee osteoarthritis (OA), while functional disability and mobility of the knee did not change. The content of the verbal suggestion was of minor importance. OLP administration may be considered as supportive analgesic treatment in elderly patients with symptomatic knee OA.

**Trial Registration:**

German Clinical Trials Register (https://www.drks.de/), DRKS00015191 (retrospectively registered).

## Introduction

Osteoarthritis (OA) is a degenerative, primarily non-inflammatory joint disease with chronic course and represents the most common joint disease among adults (1). It is characterized by functional limitations and usage-related pain (2), though there is often a mismatch between radiological stages (e.g., Kellgren degrees) and clinical complaints (3). The most influential risk factor for the development of OA of the knee is higher age. In industrialized societies the prevalence in the over 60 year’s bracket is approximately 18% (4–6). Socioeconomic burden and restrictions in quality of life are substantial (4, 7–11). Therapy of OA primarily aims at symptom reduction and prevention of disease progression and typically involves non-pharmacological interventions (e.g., weight reduction, physical training) as well as multimodal pain control strategies (12, 13). Interestingly, several studies indicate that patients with OA also benefit from placebo interventions by improving pain, stiffness, and self-reported functionality (14–16).

It has long been assumed that deception is necessary to evoke placebo effects. Accordingly, the deceptive administration of placebos is the primary subject of placebo research and is rather common in clinical practice (17–19). Nonetheless, the deceptive administration of placebos is afflicted with negative connotation and ethical concerns (20, 21). In recent years, several studies investigated the clinical effects of “non-deceptive placebos,” also referred to as “open-label placebos” (OLP). In this case, placebos are described honestly as inert substances. Interestingly, there is increasing evidence that OLP treatments go along with therapeutic benefits in various conditions, including chronic low back pain, irritable bowel syndrome, episodic migraine, allergic rhinitis, depression, attention deficit hyperactivity disorder, and cancer induced fatigue (22–30). Two recent meta-analyses concluded that even though evidence is still limited, OLPs could be a promising therapeutic approach for various clinical conditions (27, 31).

The psychological mechanisms underlying the effects of OLP administration are unknown. While the effects of deceptive placebo interventions are mediated in part by expectations raised by verbal suggestions, there is increasing evidence that conscious expectations are of limited importance for the effects of OLP (32). Furthermore, while several studies suggest that the reduction of stress and negative emotions is involved in placebo hypoalgesia (33), the role of stress reduction for the effects of OLP has not been studied.

In this randomized controlled trial, we investigated the effects of OLP administration in patients with symptomatic knee OA. In order to study the role of different verbal suggestions for the effects of OLP, we included two OLP interventions: one to “relieve pain” (“OLP-pain”) and one to “improve mood” (“OLP-mood”). The OLP-pain intervention was described as reducing pain and thereby improving health status, while the OLP-mood intervention was described as enhancing positive emotions and thereby improving health status. The rationale behind the mood-enhancing OLP intervention was based on previous studies showing that placebo interventions can improve mood (34), and that positive emotions can reduce chronic pain (35). Based on previous suggestions that conscious expectations are of limited importance for the effects of OLP (32), we hypothesized that OLP treatment would improve pain, physical functional disability, and mobility of the target knee regardless of the explicit treatment goal, i.e., to improve pain or improve mood. Secondary outcomes included health-related quality of life as well as validated stress measures in order to learn more about the role of stress reduction for OLP effects.

## Materials and Methods

### Study Design

This is a randomized controlled trial with a three-group parallel design. A total of 60 participants were randomized to one of three groups using a 1:1:1 randomization rate. After the baseline measurement on the first study day, participants were randomly assigned to no treatment (NT), OLP to reduce pain (OLP-pain), or OLP to improve mood (OLP-mood).

### Participants

Patients with pre-diagnosed painful OA of the knee were recruited *via* advertisements in local newspapers and by laying out flyers in local medical practices. Patients were included when they were ≥18 years old, in a good general/nutritional condition and were diagnosed with OA of the knee (Kellgren II–III) at least 6 months prior to the onset of the study, as evidenced by a physician letter. In addition, participants had to provide sufficient knowledge of German to understand the questionnaires, had to be able to follow the study requirements and instructions, and had to provide written informed consent. The mean pain score at the target knee had to be at least 4 on a 0–10 Numeric Rating Scale (NRS), while pain in the non-target knee should not exceed a level of 3. Exclusion criteria comprised inflammatory joint disease; other pain conditions; knee injury or surgery within the previous 3 months or planned surgery during the study period; intra-articularly injected knee pain medication; use of opioid analgesics, glucocorticoids, topical pain treatment, or systemic treatments that could affect outcomes during the study; medications that affect the autonomic nervous system or neuroendocrine system; use of psychotropic drugs; known clinical depression and/or depression score >10 on the Hospital Anxiety and Depression Scale (HADS-D) (36); drug abuse or alcoholism; pregnancy or lactation; known intolerance or allergy to lactose or gelatin; presence of malign diseases (somatic or mental) or other clinically significant conditions that, in the opinion of the study director or investigator, may preclude participation; participation in another study within the past 4 weeks.

### Procedure

Volunteers who contacted the study center received information about the study procedure and the open-label placebo intervention and were screened for the inclusion and exclusion criteria during a telephone interview. Eligible patients who consented to participate were included in the study. Study participation comprised two examinations at the Institute of Medical Psychology, LMU Munich, with a time interval of 21 days. Prior to the first study visit participants received saliva tubes along with detailed instructions on how to collect and store saliva probes the day before study visits. At both study visits, participants completed standardized questionnaires, and a 5-min electrocardiogram (ECG) was performed to assess heart rate variability (HRV). At their first visit, patients were administered a paper-and-pencil diary to monitor pain, mood, and analgesic use each day of the study period. After performing the baseline assessments at the first study visit, participants received general information on the placebo effect, namely that the placebo effect is powerful, the body can automatically respond to taking placebo pills like Pavlov’s dogs who salivated when they heard a bell, a positive attitude helps but is not necessary, and taking the pills faithfully is critical (23). Participants were then randomly assigned to NT or one of two OLP groups (OLP-pain, OLP-mood). Participants in the OLP groups received detailed information on why their treatment with OLPs was expected to be effective (Supplementary Table S1). They then obtained a medication tin with lactose capsules to be taken twice a day, thereby emphasizing the importance of regular pill intake. In order to minimize disappointment, participants in the NT group were informed about the purpose and importance of a control group in clinical trials (Supplementary Table S1). Ten days after the first study visit, all patients were contacted by phone and asked how they were feeling and whether they had any questions regarding the study, and they were thanked again for participating in the study.

### Randomization and Blinding

Computer-assisted randomization was performed by a person not involved in the experiments, who prepared sequentially numbered, sealed, and opaque randomization envelopes. Due to the open-label nature of the placebo treatment, group allocation was not blinded.

### Placebo Interventions

After treatment allocation, the participants in the two OLP groups received a box with identical gelatin capsules filled with mannitol to be taken regularly twice daily (morning and evening) for a period of 21 days. The labels of the medication boxes differed between the two OLP groups, indicating either “pain relief” or “mood improvement.” The administration of the boxes was accompanied by verbal suggestions of the effects to be expected from the respective placebo treatment. In the OLP-pain group, patients were informed that the goal of placebo administration was to reduce pain and thereby positively influence health status. In the OLP-mood group, patients were informed that the goal of placebo administration was to improve mood and thereby positively influence health status (Supplementary Table S1).

### Outcome Parameters

#### Diary and Questionnaires

Patients assessed pain and mood levels each evening of the 21-day study period using a standardized paper-and-pencil diary. Average pain/mood during the day was rated on 11-point NRS from 0 (“no pain”/”worst mood”) to 10 (“unbearable pain”/”best mood”). Patients were further asked to note the use of acute pain medication in the patient diary. At baseline and after 21 days, patients completed the following questionnaires: the *Western Ontario and McMaster Universities Osteoarthritis Index* (WOMAC) is a standardized, disease-specific self-assessment instrument with verified psychometric quality criteria (BR36a, 38). It comprises the subscales pain (range, 0–50), stiffness (range, 0–20), and physical function (range, 0–170), which are derived from 24 questions that refer to the past 2 days. Questions need to be answered on 11-point NRSs, with the left pole marked as “none” and the right pole as “extreme.” Higher scores indicate worse pain, stiffness, and physical function (39). Quality of life was assessed using the *Short Form Health Survey* (SF-36), which is a 36-item, validated patient-reported survey providing component scores for the mental and the physical health domains, with higher scores indicating better quality of life (40). Perceived stress during the past week was assessed at baseline and after 21 days using the validated *Perceived Stress Questionnaire* (PSQ-20). We report here the PSQ-20 overall score, ranging from 0 to 100, with higher values indicating higher burden (41, 42). *State anxiety* was evaluated at baseline and after 21 days using the 20-item state-anxiety subscale of the State-Trait-Anxiety Inventory (STAI), which estimates anxiety at the current moment (43, 44). The score ranges from 20 to 80, with higher scores indicating higher levels of state anxiety. Self-efficacy was assessed at baseline and after 21 days using the *General Self-Efficacy* (GSE) scale, a validated 10-item tool with good psychometric properties to measure the general, optimistic sense of perceived personal competence (45, 46). The GSE is scored 10 (minimum) to 40 (maximum self-efficacy). At the second study visit, the experimenter rated the patient’s global improvement using the *Clinical Global Impressions – Improvement (CGI-I)* scale, which uses a bipolar scaling from 1 (very much improved) to 7 (very much worse) (47).

#### Mobility of the Target Knee

The neutral-zero method was used to assess the mobility of the target knee at baseline and after 3 weeks. It is a functional measurement that describes the possible active joint mobility of an individual joint with reference to the anatomical normal (“zero”) position. With the aid of a protractor, the respective active end positions of the joint for flexion and extension are documented (48).

#### Physiological Measurements

The electrocardiogram was recorded for 5 min using the MP 150 BIOPAC System (Goleta, CA, United States) with AcqKnowledge 3.7.2 software. To increase reliability, the respiratory rate was standardized to 15 breaths per minute using a metronome (49). The ECG signal was sampled at a rate of 500 Hz (50). Intervals between successive R peaks (RR intervals) were extracted from the electrocardiogram signal using the peak-detection function implemented in AcqKnowledge 3.7.2. RR-time series were examined and screened for artifacts based on the procedure developed by Porges and Byrne (51), and then subjected to Kubios HRV software version 2.2 (Kuopio, Finland) to calculate the power of the high frequency band (0.15–0.4 Hz) of HRV relative to the total power (0–0.4 Hz). HRV is a measure of cardiac vagal activity and is used to estimate cardiovascular stress, with lower values indicating higher stress (52).

Salivary cortisol samples were collected at the day before study examinations at standardized daytimes [08:00 a.m., 00:00 a.m., 05:00 p.m., 09:00 p.m.; (53)] by using commercially available cotton swabs (Salivette^®^, Sarstedt, Nümbrecht, Germany). Participants were instructed to chew the swabs for at least 60 s before storing it back into a tube, and to hand out the four salivary tubes to the study personnel at each examination day. Saliva samples were centrifuged at 2000 rpm at 4°C and stored at −20°C until analysis. Salivary cortisol concentration was assessed using cortisol saliva assay kits from IBL International GMBH (RE52611). All saliva samples were analyzed in duplicate following the manufacturer’s protocol. The area under the curve (AUC cortisol) was calculated for each examination day according to the trapezoid rule as outlined by Pruessner et al. (54). AUC cortisol reflects the overall secretory activity of the humoral stress axis throughout the day.

### Primary and Secondary Outcome Parameters and Sample Size Calculation

Pre-specified primary outcomes included group differences in improvement of knee pain and function (NRS pain, WOMAC) and range of mobility (neutral-zero method) from baseline to follow-up at 3 weeks. Secondary outcomes comprised the course of pain and mood ratings (NRS) and the need for analgesics during the 3-week study period, global clinical improvement (CGI-I) after 21 days, and pre–post changes in physical and mental quality of life (SF-36), perceived stress (PSQ-20), diurnal salivary cortisol (AUC cortisol), and HRV. We further evaluated pre–post changes in state anxiety (STAI-state) and self-efficacy (GSE).

Sample size calculation was performed for the primary outcome WOMAC pain, namely the differences in improvement of WOMAC pain between the OLP and NT groups (randomization rate 2:1). We estimated that a total sample size of 60 would provide 80% power (one-sided *p* < 0.05) to detect a moderate-to-large effect (*d* = 0.7), as reported by Kaptchuk et al. (23). Sample size calculation was performed using GPower (version 3.1).

### Statistical Analyses

Before analysis, the normality assumption was tested for all continuous outcome parameters using normal probability plots of the residuals, while the homoscedasticity assumption was checked using the Levene test and normal Q–Q plots. Because the daily ratings did not fulfill the normality assumption, available pain and mood ratings (5.9% missing values) were averaged for each week. All pre–post changes of continuous outcomes fulfilled the one-way analysis of variance (ANOVA) assumptions and were subjected to ANOVAs, with “group” (NT, OLP-pain, OLP-mood) as the between-subject factor. We primarily evaluated the contrasts between the NT and OLP groups (one-tailed) to test whether OLP has beneficial effects compared to NT. In an exploratory approach, we also evaluated the contrasts between the OLP-pain and OLP-mood groups (two-tailed). Pre–post changes in knee flexion and knee extension as well as post-treatment CGI-I scores were evaluated using Mann–Whitney *U*-tests, again contrasting NT vs. OLP groups (one-tailed) and OLP-pain vs. OLP-mood groups (two-tailed). Cohen’s *d* effects sizes were calculated for parametric and non-parametric statistics (55), with 0.2 defined as small, 0.5 as medium, and 0.8 as large effect size (56). Statistical analyses were performed using IBM SPSS Statistics 25. For all statistical tests, a significance level of α = 0.05 was assumed.

## Results

### Sample Characteristics

**FIGURE 1:**
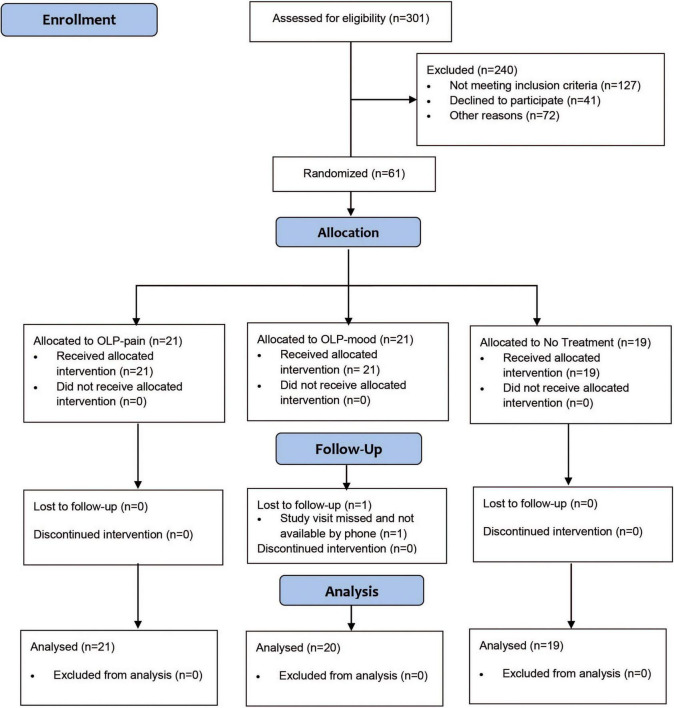
Flow chart.

Recruitment took place between November 2016 and September 2017. A total of 61 out of 261 patients who contacted the study center were enrolled in the study; reasons for exclusion are summarized in Figure 1. One patient was lost to follow-up after the baseline visit (he/she did not show up for the second study visit and could not be reached by phone). A total of 60 patients completed the study and were included in the analyses. None of the participants had taken part in a placebo study before. At baseline, the three groups were comparable in terms of sociodemographic and clinical characteristics, except for knee extension, which was significantly larger in the NT group (Table 1).

**TABLE 1:** Demographic and clinical characteristics at baseline.

Variable	OLP pain (*n* = 21)	OLP mood (*n* = 20)	NT (*n* = 19)	*p*-Value
Age (years), mean (SD)	64.19 (9.3)	66.8 (9.7)	69.84 (9.63)	0.183^1^
Sex (female/male), *n*	12/9	9/11	12/7	0.507^2^
Baseline pain (NRS), mean (SD)	2.67 (1.85)	2.58 (2.04)	2.83 (2.18)	0.927^1^
Baseline mood (NRS), mean (SD)	6 (2.6)	6.3 (2)	5.8 (2.8)	0.834^1^
WOMAC, mean (SD)				
Pain	23.05 (7.65)	23.1 (8.14)	21.37 (8.02)	0.742^1^
Stiffness	10.43 (4.44)	9.2 (3.02)	10.61 (4.79)	0.466^1^
Functionality	79.26 (31.09)	72.65 (28.27)	72.79 (32.03)	0.732^1^
Knee mobility, median (IQR)				
Knee flexion (°)	90 (88.5; 110)	100 (88.5; 109.8)	91 (81; 100)	0.592^3^
Knee extension (°)	0 (0; 0)	0 (0; 0)	0 (0; 4)	<0.001^3^

*^1^T-test. ^2^Chi-Quadrat test. ^3^Kruskal–Wallis test.*

*OLP, open-label placebo; WOMAC, Western Ontario and McMaster Universities Osteoarthritis Index.*

### Patient Diary

**FIGURE 2:**
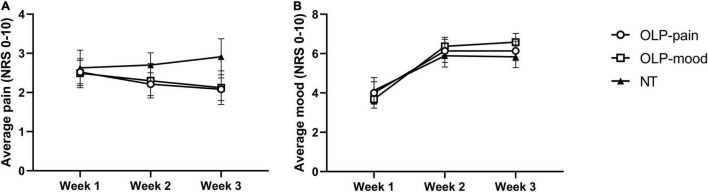
Weekly averages of daily NRS pain ratings **(A)** and NRS mood ratings **(B)**. Data are displayed as means ± SE.

From week 1 to week 3, NRS pain ratings decreased in the OLP-pain and OLP-mood groups and increased in the NT group (Figure 2A and Table 2). Contrast analyses indicated a larger reduction in NRS pain from week 1 to week 3 in the combined OLP groups compared to the NT group [*t*(56) = 2.282, *p* = 0.013, *d* = 0.64], while changes in the two OLP groups did not differ significantly from each other [*t*(56) = 0.182, *p* = 0.856, *d* = 0.05]. The need for analgesics remained stable in the three treatment groups (Table 2).

**TABLE 2:** Outcome parameters at baseline and after 3 weeks.

Variable	OLP pain (*n* = 21)			OLP mood (*n* = 20)			NT (*n* = 19)			Cohen’s *d*	
							
	Pre	Post	Mean change (95% CI) or median change (IQR)	Pre	Post	Mean change (95% CI)	Pre	Post	Mean change (95% CI) or median change (IQR)	OLP vs. NT	OLP-pain vs. OLP-mood
**Patient diary (week 1 → week 3)**											
NRS pain, mean (SD)	2.52 (1.38)	2.08 (1.32)	−0.37 (−0.75; 0.01)	2.49 (1.61)	2.12 (1.86)	−0.37 (−0.75; 0.01)	2.63 (1.97)	2.91 (2)	0.28 (−0.29; 0.86)	0.64*	*0.05*
NRS mood, mean (SD)	4 (2.61)	6.14 (2.29)	2.14 (0.01; 4.28)	3.68 (1.97)	6.58 (1.92)	2.89 (1.06; 4.73)	4.17 (2.79)	5.84 (2.41)	1.94 (−0.32; 4.2)	*0.13*	*0.15*
Patients using analgesics during study period, *n*	5	5		5	5		6	6		–	–
Number of days, median (IQR)	2 (0.5; 4.5)	1 (0; 3)	−1 (−2; 0)	1 (0; 5.5)	1 (0.5; 4)	0 (−1.5; 5)	0.5 (0; 0.25)	1 (0; 1.25)	−0.5 (−1.25; 1.25)	*0.11*	*0.35*
**WOMAC (baseline → follow-up)**											
WOMAC pain, mean (SD)	23.05 (7.65)	17.57 (7.54)	−5.48 (−10.53; −0.42)	23.1 (8.14)	18.8 (8.91)	−4.3 (−6.66; −1.94)	21.37 (8.02)	20.68 (8.76)	−0.68 (−4.15; 2.78)	0.55*	*0.17*
WOMAC stiffness, mean (SD)	10.43 (4.44)	7.81 (4.07)	−2.62 (−4.93; −0.31)	9.2 (3.02)	8.5 (3.32)	−0.7 (−2.24; 0.84)	10.74 (4.69)	9.63 (4.47)	−1.11 (−2.59; 0.38)	*0.14*	*0.42*
WOMAC function, mean (SD)	79.26 (31.09)	59.67 (24.38)	−19.59 (−35.34; −3.84)	72.65 (28.27)	64.55 (30.68)	−8.1 (−15.29; −0.92)	72.79 (32.03)	67.47 (30.2)	−5.32 (−15.21; 4.58)	*0.34*	*0.4*
**Knee mobility (baseline → follow-up)**											
Knee flexion (°), median (IQR)	90 (88.5; 110)	90 (81; 97.5)	−7 (−20.5; 10.5)	100 (88.5; 109.8)	96 (90; 110)	0 (−0.8; 5)	91 (81; 100)	92 (85; 102)	0 (−9; 8)	*0.03*	*0.25*
Knee extension (°), median (IQR)	0 (0; 0)	0 (0; 0)	0 (0; 0)	0 (0; 0)	0 (0; 0)	0 (0; 0)	0 (0; 4)	0 (2; 2)	0 (0; 2)	*0.09*	*0.09*
**Clinical improvement (follow-up)**											
CGI improvement, median (IQR)	–	2 (2; 3)	–	–	2.5 (2; 3)	–	–	3 (3; 3)	–	0.59*	*0.03*
**Quality of life (baseline → follow-up)**											
SF-36 MCS, mean (SD)	31.28 (10.3)	34.87 (8.74)	2.05 (−2.78; 6.88)	37.88 (8.35)	40.14 (8.99)	0.58 (−2.76; 3.91)	33.1 (0.62)	36.64 (10.27)	3.17 (−0.19; 6.53)	*0.24*	*0.11*
SF-36 PCS, mean (SD)	52.79 (10.61)	54.83 (8.76)	3.59 (−1; 8.12)	50.93 (11.3)	54.1 (9.22)	2.26 (−0.65; 5.17)	52.32 (9.24)	52.9 (9.75)	3.54 (−0.19; 7.28)	*0.08*	*0.14*
**Stress (baseline → follow-up)**											
PSQ-20, mean (SD)	28.83 (14.49)	23.92 (14.95)	−4.3 (−9.06; 0.46)	25.74 (13.16)	22.89 (15.61)	−1.08 (−6.41; 4.26)	24.9 (14.95)	21.05 (12.85)	−4.22 (−7.56; −0.87)	*0.17*	*0.3*
AUC cortisol (ln + 1), mean (SD)	0.71 (0.31)	0.69 (0.36)	−0.02 (−0.24; 0.19)	0.88 (0.38)	0.8 (0.36)	−0.94 (−0.34; 0.16)	0.82 (0.43)	0.83 (0.44)	0.01 (−0.13; 0.15)	*0.16*	*0.14*
HRV (%), mean (SD)	45.96 (14.17)	45.35 (18.46)	−0.21 (−10.64; 9.43)	50.74 (23.89)	50.8 (19.17)	0.06 (−11.81; 11.94)	40.81 (18.08)	47.21 (21.18)	6.4 (−7.05; 19.86)	*0.27*	*0.02*
**Further (baseline → follow-up)**											
STAI-State, mean (SD)	33.62 (8.03)	34.05 (9.4)	0.43 (−2.04; 2.89)	31.8 (6.9)	32.25 (8.45)	0.45 (−2.23; 3.13)	31.42 (6.15)	30.47 (4.61)	−0.95 (−3.09; 1.2)	*0.26*	*0*
GSE, mean (SD)	32.9 (5.66)	32.48 (5.75)	−0.43 (−2.95; 2.1)	31.6 (5.21)	31.15 (6.15)	−0.45 (−4.1; 3.2)	31.89 (3.56)	33.89 (3.4)	2 (−0.11; 4.11)	*0.4*	*0*

*OLP, open-label placebo; NT, no treatment; NRS, Numeric Rating Scale; SD, standard deviation; md, median; WOMAC, Western Ontario and McMaster Universities Osteoarthritis Index; CGI, clinical global impression; SF, short form health survey; MCS, mental component score; PCS, physical component score; PSQ, perceived stress questionnaire; AUC, area under the curve; HRV, heart rate variability; STAI, State-Trait-Anxiety Inventory; HR, heart rate; GSE, General Self-Efficacy.*

*p < 0.05.

From week 1 to week 2, NRS mood ratings increased in the three groups to a similar extent and remained stable thereafter (Figure 2B and Table 2). Planned contrasts revealed no differences in mood improvement from week 1 to week 3 between the OLP and NT groups [*t*(55) = −0.462, *p* = 0.323, *d* = 0.13], nor between the two OLP groups [*t*(55) = 0.543, *p* = 0.590, *d* = 0.15].

### Western Ontario and McMaster Universities Osteoarthritis Index Questionnaire

The group means of the WOMAC subscores pain, stiffness, and function before and after the intervention as well as pre–post changes are shown in Table 2. Contrast analyses indicated a greater reduction in WOMAC pain from baseline to follow-up in the combined OLP groups compared with the NT group [*t*(57) = 1.835, *p* = 0.036, *d* = 0.55]. Changes in the two OLP groups did not differ from each other [*t*(57) = 0.456, *p* = 0.65, *d* = 0.17].

The WOMAC subscores function and stiffness did not differ between the OLP and NT groups [function: *t*(57) = 1.223, *p* = 0.226, *d* = 0.34; stiffness: *t*(57) = 0.505, *p* = 0.308, *d* = 0.14] or between the two OLP groups [function: *t*(57) = 1.259, *p* = 0.182; stiffness: *t*(57) = 1.552, *p* = 0.126, *d* = 0.17].

### Knee Mobility and Global Clinical Improvement

The mobility of the target knee as assessed by the neutral-zero method did not change differently between the OLP and NT groups (flexion: Mann–Whitney *U*-test, *z* = −0.119, *p* = 0.453, *d* = 0.03; extension: *z* = −0.475, *p* = 0.317, *d* = 0.09), nor between the OLP-pain and OLP-mood groups (flexion: *z* = −0.797, *p* = 0.213, *d* = 0.25; extension: *z* = −0.607, *p* = 0.272, *d* = 0.09; Table 2).

Global clinical improvement on the CGI-I scale was larger in the OLP group than in the NT group (Mann–Whitney *U*-test, *z* = −2.457, *p* = 0.007, *d* = 0.59), whereas there was no difference between the OLP-pain and OLP-mood groups (*z* = −0.114, *p* = 0.910, *d* = 0.03; Table 2).

### Quality of Life

The pre–post changes in mental quality of life (MCS; SF-36) remained unaffected by OLP treatment [OLP vs. NT groups, *t*(57) = −0.865, *p* = 0.170, *d* = 0.24; OLP-pain vs. OLP-mood groups, *t*(57) = 0.425, *p* = 0.673, *d* = 0.11; Table 2]. Similarly, the pre–post changes in physical quality of life (PCS) did not differ between groups [OLP vs. NT, *t*(57) = 0.270, *p* = 0.394, *d* = 0.08; OLP-pain vs. OLP-mood, *t*(57) = −0.517, *p* = 0.607, *d* = 0.14; Table 2].

### Stress Parameters

Perceived stress (PSQ-20) did not change differently between groups [OLP vs. NT, *t*(50) = 0.569, *p* = 0.261, *d* = 0.17; OLP-pain vs. OLP-mood, *t*(50) = 1.058, *p* = 0.295, *d* = 0.3; Table 2]. Also diurnal salivary cortisol excretion (AUC cortisol) was unaffected by OLP treatment [OLP vs. NT, *t*(55) = 0.586, *p* = 0.28, *d* = 0.16; OLP-pain vs. OLP-mood, *t*(55) = −0.507, *p* = 0.614, *d* = 0.14; Table 2], as was HRV [OLP vs. NT, *t*(55) = 0.959, *p* = 0.171, *d* = 0.27; OLP-pain vs. OLP-mood, *t*(55) = 0.084, *p* = 0.933, *d* = 0.02; Table 2].

### State Anxiety and Self-Efficacy

State anxiety did not change differently between the OLP and the NT groups [*t*(57) = −0.953, *p* = 172, *d* = 0.26], nor between the OLP-pain and OLP-mood groups [*t*(57) = 0.013, *p* = 0.990, *d* = 0; Table 2]. OLP did not affect self-efficacy [OLP vs. NT, *t*(57) = 1.444, *p* = 0.057, *d* = 0.4; OLP-pain vs. OLP-mood, *t*(57) = −0.011, *p* = 0.991, *d* = 0; Table 2].

## Discussion

In this randomized controlled trial, we examined the effects of OLP administration accompanied by two different verbal suggestions on symptomatic OA of the knee. Results revealed that OLP administration significantly reduced knee pain, regardless of whether patients were informed that the placebo would “decrease pain” or “improve mood.” In addition, clinical global impression was improved in the OLP groups compared to the NT group. Our results confirm previous findings that OLP treatment can improve chronic pain (57) and extend them to typically elderly patients with symptomatic OA of the knee. We found no effect of OLP administration on patient-reported functional disability of the knee and observer-reported mobility.

While the mean age of patients in the previous studies ranged between 40 and 60 years (23, 25, 30, 58), our results suggest that also older patients can be successfully treated by OLP. The acceptance of OLP treatment in the target population was sufficiently high, with 106 out of 261 patients declining to participate for various reasons, among them 20 patients who wanted active treatment. The acceptance of OLP treatment in the general population is generally high. For example, a survey in the United States revealed that 85% of the respondents considered OLP administration acceptable for patients with chronic abdominal pain (59).

We administered two OLP treatments differing by their declared goal, namely pain relief or mood improvement. Results show that neither pain or mood ratings nor any of the other outcomes differed between the two placebo groups. These results may partially be due to the small sample size of our study, with limited statistical power to detect differential effects between the two OLP interventions. For example, the respective effect sizes for the comparison of the WOMAC subscores stiffness and function scores were moderate at 0.4, suggesting that larger sample sizes would have been necessary to detect significant differences. However, with regard to pain, the effect sizes for the differences between the two OLP groups were generally small. Thus, our results suggest that OLP treatment was effective regardless of whether the explicit goal was to improve pain, or mood. Although we did not assess treatment expectations in our study, the finding that suggestion-specific effects did not occur indirectly supports previous findings that expectations play a limited role in OLP treatment. In a qualitative study, for example, patients receiving OLP treatment denied having positive expectations (32). Furthermore, an OLP study in patients with hot flushes showed that the increase in positive expectations after OLP administration was unrelated to clinical improvements (60). However, instead of OLP-specific expectations, more general treatment expectations may play a role: a recent study comparing OLP acupuncture with OLP pills for the relief of experimental pain reported that expectations toward OLP treatments did not predict the placebo analgesic effect, whereas general expectations toward (active) acupuncture did (61). The hypoalgesic effects of OLP treatment in our study may likewise be related to positive expectations toward pharmacological drugs rather than OLP treatment.

Recent Bayesian brain models offer an alternative way to explain OLP effects, apart from positive expectations (32, 57, 62). In these models, perception is viewed as a process of prediction based on the integration of sensory input, prior experience, and contextual cues. Any discrepancy between the predicted and the actual sensory input will result in a prediction error, which can be resolved in one of three ways: the prediction model can be updated, the sensory input can be attenuated, or the sensory input can be amplified. According to this model, a placebo analgesic effect results from the attenuation of the sensory input. In the case of deceptive placebo administration, this is most probably due to positive expectations, which lower the level of predicted pain and thereby pain perception. In the case of OLP treatment, the attenuation of perceived pain could be primarily due to reduced precision of the predicted pain signal, i.e., increased uncertainty, resulting from the paradox information of receiving “substances that have no active ingredients” (57).

The question of whether OLP treatment can improve health-related quality of life remains unclear, as previous studies have shown mixed results. Disease-specific quality-of-life instruments appear to be better suited to demonstrate the beneficial effects of OLP (23, 28, 60) than more general instruments such as the SF-36 (26, 63, 64). Also in our study, OLP led to improvement in the pain subscale of the disease-specific WOMAC questionnaire, whereas the mental and physical components of health-related quality of life as assessed by the SF-36 remained unaffected. It should be mentioned that the observation period of 3 weeks may have been too short to capture positive effects of OLP treatment on mental and physical health-related quality of life. Gradual increase in physical activity due to reduced pain could lead to improved muscle strength over longer time periods, which might result in improved quality of life at a later time. Indeed, studies reporting positive treatment effects on health-related quality of life in patients with knee OA typically comprise longer observation periods (65). Our finding that OLP treatment did not improve functional disability of the knee, as assessed by the WOMAC subscores function and stiffness, is contrasts two recent OLP studies, which showed improvement of pain and functional disability in patients with chronic low back pain (25, 30). Again, the sample size of our study may have been too small to detect subtle changes in functional disability with OLP treatment (*d* = 0.34). Regarding objective knee mobility, our findings are consistent with those of Kleine-Borgmann et al. (30), who also reported no effect of OLP administration on objective spine mobility.

Finally, we explored whether the beneficial effects of OLP may be due to the reduction of stress and negative emotions. Study results consistently argue against such a view, as perceived stress, state anxiety and physiological stress parameters were not affected by OLP administration. Similarly, Kleine-Borgmann et al. (30) reported no changes in stress and anxiety after OLP treatment in patients with chronic back pain. However, in their study, OLP administration reduced (non-clinical) depression scores, whereas in our study, OLP had no effect on mood ratings. This discrepancy may be due to the use of a single-item NRS in our study, which has been shown to correlate well with depression scales in clinical populations (66), but this may not be true for non-psychiatric patients. Alternatively, the mood improvement in the NT group may have masked the mood-enhancing effect of OLP administration. Improved mood in the NT group may best be explained by the Hawthorne effect, i.e., an improvement due to additional attention by study personnel and the knowledge of being under observation (67).

Several possible limitations of the study have to be mentioned. The sample size of our study was rather small and some of the beneficial effects of OLP in patients with OA of the knee may have been missed due to the lack of statistical power. Nonetheless, the reported effect sizes provide a solid empirical basis to design future OLP studies in patients with symptomatic OA of the knee. Furthermore, the assessment of observer-reported outcomes was not blinded and the improvement in the CGI-I scale by OLP administration should be interpreted with caution. In addition, the study research team’s appreciative attention may have blurred some of the beneficial effects of OLP treatment, particularly on mood. Furthermore, our exclusion criteria were rather strict and many patients were excluded because of various illnesses or medication. This limits the external validity of our results, especially with respect to elderly people who frequently have multiple diseases. Finally, the short duration of OLP treatment does not allow to draw conclusions about the potential value of OLP treatment in clinical practice. However, Carvalho et al. (68) recently published a 5-year follow-up of a randomized controlled trial on OLP in patients with chronic low back pain, suggesting that the improvements in pain and disability after OLP are long lasting.

In conclusion, our study is the first to provide evidence that elderly patients with symptomatic OA of the knee show pain relief by OLP treatment. Results lend support to the notion that concealment and deception are not necessary to evoke placebo effects in patients with chronic pain conditions. Future studies should address the role of synergistic and opposite verbal suggestions for OLP effects, as well as the long-term effects of OLP administration and its acceptability and feasibility in clinical practice.

## Data Availability Statement

The datasets generated and/or analyzed during the current study are available from the corresponding author on request.

## Ethics Statement

The studies involving human participants were reviewed and approved by the Ethics Committee of the Medical Faculty, LMU Munich, Munich, Germany. The patients/participants provided their written informed consent to participate in this study.

## Author Contributions

SS and KM designed the study. EO, SS, and AH carried out the experiment. EO, SS, AH, FR, and MM analyzed the raw data. SF and KM supervised the data analyses. EO, SS, and KM performed the statistical analyses. EO and KM wrote the first draft of the manuscript. All authors contributed to manuscript revision, read, and approved the submitted version.

## Conflict of Interest

The authors declare that the research was conducted in the absence of any commercial or financial relationships that could be construed as a potential conflict of interest.

## Publisher’s Note

All claims expressed in this article are solely those of the authors and do not necessarily represent those of their affiliated organizations, or those of the publisher, the editors and the reviewers. Any product that may be evaluated in this article, or claim that may be made by its manufacturer, is not guaranteed or endorsed by the publisher.
