# Supplementary material for: Open-Label Placebo Administration Decreases Pain in Elderly Patients With Symptomatic Knee Osteoarthritis – A Randomized Controlled Trial
Source: Front Psychiatry. 2022 May 6;13:853497. doi: 10.3389/fpsyt.2022.853497 (PMC9122028; doi:10.3389/fpsyt.2022.853497)
Supplement: Supplementary file 1 [file Table_1.pdf]

SUPPLEMENTARY MATERIAL

Table S1: Instructions according to study group (translated from German).

**Pain improvement group (OLP-pain)**<br><br>You are in the placebo group to improve pain. The treatment goal is to reduce your pain and thereby induce a positive effect on your current health state. This effect has already been scientifically proven in previous placebo studies and is real, not imagery. You will receive a medicine box, which is filled with capsules. As already mentioned, these are placebos, which have no pharmacological effect. We ask you to take one capsule twice a day (in the morning and in the evening) for the whole duration of the study (21 days). It is of great importance to take the capsules regularly. If you feel improvements/changes during the study, they are not imagery. To track your health progress, you will be given a study diary to keep with you. Please enter your daily pain and mood levels every evening in the diary.
**Mood improvement group (OLP-mood)**<br><br>You are in the placebo group to improve mood. The treatment goal is to improve your mood and thereby induce a positive effect on your current health state. This effect has already been scientifically proven in previous placebo studies and is real, not imagery. You will receive a medicine box, which is filled with capsules. As already mentioned, these are placebos, which have no pharmacological effect. We ask you to take one capsule twice a day (in the morning and in the evening) for the whole duration of the study (21 days). It is of great importance to take the capsules regularly. To track your health progress, you will be given a study diary to keep with you. Please enter your daily pain and mood levels every evening in the diary.
**No treatment group (NT)**<br><br>You are in the control group. The control group receives no treatment, but is important to control for natural variations in health status. Without a control group, the study would not be evaluable and we would not be able to obtain clear scientific results. This means that your participation is of great importance to our study. To track your health progress, you will be given a study diary to keep with you. Please enter your daily pain and mood levels every evening in the diary.
